# Supplementary material for: Effects of Ginger Intake on Chemotherapy-Induced Nausea and Vomiting: A Systematic Review of Randomized Clinical Trials
Source: Nutrients. 2022 Nov 23;14(23):4982. doi: 10.3390/nu14234982 (PMC9739555; doi:10.3390/nu14234982)
Supplement: Supplementary file 1 [file nutrients-14-04982-s001.zip › nutrients-1983824-supplementary.pdf]

Supplementary Table S1. Search strategy

	Search term
	**PubMed (search date: no date limit – 5/11/2021)**
**1**	ginger[MeSH] OR ginger*[tiab] OR ‘zingiber officinale’*[tiab] OR ‘officinales, zingiber’[tiab]
**2**	neoplasms[MeSH] OR ‘chemotherapy, adjuvant’[MeSH] OR ‘consolidation chemotherapy’[MeSH] OR ‘induction chemotherapy’[MeSH] OR photochemotherapy[MeSH] OR ‘maintenance chemotherapy’[MeSH] OR ‘chemotherapy, cancer, regional perfusion’[MeSH] OR ‘antineoplastic combined chemotherapy protocols’[MeSH] OR ‘electrochemotherapy’[MeSH] OR neoplas*[tiab] OR tumor*[tiab] OR tumour*[tiab] OR malignanc*[tiab] OR cancer*[tiab] OR chemotherap*[tiab] OR ‘antineoplastic combined chemotherapy regimens’ [tiab] OR ‘antineoplastic chemotherapy protocol’*[tiab] OR ‘chemotherapy protocol, antineoplastic’ [tiab] OR ‘protocol, antineoplastic chemotherapy’ [tiab] OR ‘cancer chemotherapy protocol’*[tiab] OR ‘protocol, cancer chemotherapy’ [tiab] OR ‘adjuvant chemotherapy’ [tiab] OR ‘consolidation chemotherapies’ [tiab] OR ‘chemotherapy, consolidation’ [tiab] OR ‘regional perfusion antineoplastic chemotherapy’ [tiab] OR ‘isolation perfusion cancer chemotherapy’ [tiab] OR ‘cancer chemotherapy, regional perfusion’ [tiab] OR ‘perfusion cancer chemotherapy, regional’ [tiab] OR ‘regional perfusion cancer chemotherapy’ [tiab] OR electrochemotherapies OR ‘chemotherapy, induction’ [tiab] OR ‘maintenance chemotherapies’[tiab] OR photochemotherapies[tiab]
**3**	nausea[MeSH] OR vomiting [MeSH] OR emetics[MeSH] OR Antiemetics[MeSH] OR emesis[tiab] OR emetogenic[tiab] OR emetogenicity[tiab] OR nausea[tiab] OR nauseous[tiab] OR vomit*[tiab] OR emetic*[tiab] OR regurgit*[tiab] OR ‘chemotherapy induced nausea and vomiting’ [tiab] OR ‘chemotherapy-induced nausea and vomiting’ [tiab] OR CINV[tiab]
**4**	‘Randomized controlled trial’[pt] OR ‘controlled clinical trial’[pt] OR ‘clinical study’[pt] OR ‘clinical trial’[pt] OR ‘comparative study’[pt] OR randomized[tiab] OR randomised[tiab] OR placebo[tiab] OR randomly[tiab] OR trial[tiab] OR groups[tiab] OR ‘Single blind’[tiab] OR ‘Double blind’[tiab] OR intervention[tiab]
**5**	#1 AND #2 AND #3 AND #4

	Search term
	**EMBASE (search date: no date limit – 5/11/2021)**
**1**	ginger/exp OR ginger*:ti,ab OR 'zingiber officinale*':ti,ab OR 'officinales, zingiber':ti,ab
**2**	neoplasms/exp OR 'chemotherapy, adjuvant'/exp OR 'consolidation chemotherapy'/exp OR 'induction chemotherapy'/exp OR 'photochemotherapy'/exp OR 'maintenance chemotherapy'/exp OR 'chemotherapy, cancer, regional perfusion'/exp OR 'antineoplastic combined chemotherapy protocols'/exp OR 'electrochemotherapy'/exp OR neoplas*:ti,ab OR tumor*:ti,ab OR tumour*:ti,ab OR malignanc*:ti,ab OR cancer*:ti,ab OR chemotherap*:ti,ab OR 'antineoplastic combined chemotherapy regimens':ti,ab OR 'antineoplastic chemotherapy protocol*':ti,ab OR 'chemotherapy protocol, antineoplastic':ti,ab OR 'protocol, antineoplastic chemotherapy':ti,ab OR 'cancer chemotherapy protocol*':ti,ab OR 'protocol, cancer chemotherapy':ti,ab OR 'adjuvant

	chemotherapy':ti,ab OR 'consolidation chemotherapies':ti,ab OR 'chemotherapy, consolidation':ti,ab OR 'regional perfusion antineoplastic chemotherapy':ti,ab OR 'isolation perfusion cancer chemotherapy':ti,ab OR 'cancer chemotherapy, regional perfusion':ti,ab OR 'perfusion cancer chemotherapy, regional':ti,ab OR 'regional perfusion cancer chemotherapy':ti,ab OR electrochemotherapies:ti,ab OR 'chemotherapy, induction':ti,ab OR 'maintenance chemotherapies':ti,ab OR photochemotherapies:ti,ab OR 'chemotherapy induced nausea and vomiting':ti,ab OR 'chemotherapy-induced nausea and vomiting':ti,ab OR cinv:ti,ab
**3**	nausea/exp OR vomiting/exp OR emetics/exp OR antiemetics/exp OR emesis:ti,ab OR emetogenic:ti,ab OR emetogenicity:ti,ab OR nausea:ti,ab OR nauseous:ti,ab OR vomit*:ti,ab OR emetic*:ti,ab OR regurgit*:ti,ab
**4**	'randomized controlled trial':pt OR 'controlled clinical trial':pt OR 'clinical study':pt OR 'clinical trial':pt OR 'comparative study':pt OR randomized:ti,ab OR randomised:ti,ab OR placebo:ti,ab OR randomly:ti,ab OR trial:ti,ab OR groups:ti,ab OR 'single blind':ti,ab OR 'double blind':ti,ab OR intervention:ti,ab
**5**	#1 AND #2 AND #3 AND #4

Search term	
**Web of Science (search date: no date limit – 5/11/2021)**	
**1**	TS=(ginger OR ginger* OR "zingiber officinale*" OR "officinales, zingiber")
**2**	TS=(neoplasms OR "chemotherapy, adjuvant" OR "consolidation chemotherapy" OR "induction chemotherapy" OR photochemotherapy OR "maintenance chemotherapy" OR "chemotherapy, cancer, regional perfusion" OR "antineoplastic combined chemotherapy protocols" OR electrochemotherapy OR neoplas* OR tumor* OR tumour* OR malignanc* OR cancer* OR chemotherap* OR "antineoplastic combined chemotherapy regimens" OR "antineoplastic chemotherapy protocol*" OR "chemotherapy protocol, antineoplastic" OR "protocol, antineoplastic chemotherapy" OR "cancer chemotherapy protocol*" OR "protocol, cancer chemotherapy" OR "adjuvant chemotherapy" OR "consolidation chemotherapies" OR "chemotherapy, consolidation" OR "regional perfusion antineoplastic chemotherapy" OR "isolation perfusion cancer chemotherapy" OR "cancer chemotherapy, regional perfusion" OR "perfusion cancer chemotherapy, regional" OR "regional perfusion cancer chemotherapy" OR electrochemotherapies OR "chemotherapy, induction" OR "maintenance chemotherapies" OR photochemotherapies OR "chemotherapy induced nausea and vomiting" OR "chemotherapy-induced nausea and vomiting" OR CINV)
**3**	TS=(nausea OR vomiting OR emetics OR Antiemetics OR emesis OR emetogenic OR emetogenicity OR nausea OR nauseous OR vomit* OR emetic* OR regurgit*)
**4**	TS=("Randomized controlled trial" OR "controlled clinical trial" OR "clinical study" OR "clinical trial" OR "comparative study" OR randomized OR randomised OR placebo OR randomly OR trial OR groups OR "Single blind" OR "Double blind" OR intervention)
**5**	#1 AND #2 AND #3 AND #4
